# Prevalence and risk factors of postmenopausal osteoporosis: a systematic review and meta-analysis based on multivariate logistic regression

**DOI:** 10.3389/fendo.2026.1950298

**Published:** 2026-09-29

**Authors:** Zhao Qin, Jun Liao, Xiaoming Peng, Yilin Teng, Baicheng Wan, Shaohui Zong

**Affiliations:** 1 Department of Medical Cosmetology, The First Affiliated Hospital of Guangxi Medical University, Nanning, China; 2 Department of Spine Osteopathia, The First Affiliated Hospital of Guangxi Medical University, Nanning, China; 3 Department of Joint and Bone Diseases, Yuebei People’s Hospital, Affiliated Hospital of Shantou University, Shaoguan, China; 4 Wuming Hospital of Guangxi Medical University, Nanning, China

**Keywords:** meta-analysis, osteoporosis, postmenopausal women, prevalence, risk factors

## Abstract

**Objective:**

This meta-analysis aimed to investigate the prevalence and risk factors for Postmenopausal Osteoporosis (PMO) to provide a basis for early clinical identification of high-risk populations.

**Methods:**

We systematically searched English databases (PubMed, Cochrane Library, Embase, Web of Science) and Chinese databases (CNKI, Wanfang Data, VIP Database, Sinomed) up to May 2026 for observational studies using multivariate logistic regression to analyze risk factors for PMO. The meta-analysis was conducted following the PRISMA guidelines. After data extraction, pooled odds ratios (OR) with 95% confidence intervals (CI) were calculated using STATA 15.0.

**Results:**

A total of 37 studies involving 32,366 postmenopausal women were included. Among these, 36 studies reported the prevalence of osteoporosis, yielding an overall prevalence of 33.87%. The meta-analysis identified the following factors as significantly associated with an increased risk of PMO: Age (OR = 1.16); Postmenopausal duration (OR = 1.23); Postmenopausal duration > 10 years (OR = 2.81); Number of births (OR = 1.32); With ≥ 2 births (OR = 3.07); Smoking (OR = 1.55); Lack of exercise (OR = 1.66); History of previous fracture (OR = 1.86); Diabetes mellitus (OR = 2.24); Conversely, a higher body mass index (BMI) (OR = 0.80), older age at menopause (OR = 0.92), and estrogen replacement therapy (OR = 0.45) were identified as protective factors. No significant association was found for the following factors: Family history of osteoporosis; Regular exercise; Hypertension; Calcium supplementation.

**Conclusion:**

Osteoporosis is highly prevalent among postmenopausal women, particularly within the East Asian cohorts that comprised the majority of this meta-analysis. Several independent risk factors were identified, including age, longer postmenopausal duration, multiparity, smoking, physical inactivity, previous fracture, and diabetes. Estrogen replacement therapy showed a strong protective effect. While high statistical heterogeneity for several factors was addressed using random-effects models and sensitivity analyses, these findings—derived predominantly from Chinese cohorts—should be interpreted with consideration of this variability. Nevertheless, they provide a robust framework for early risk stratification and targeted prevention strategies in this high-risk population.

**Systematic review registration:**

https://www.crd.york.ac.uk/prospero/, identifier CRD420251274575.

## 1 Introduction

Osteoporosis is a systemic skeletal disorder characterized by compromised bone strength, predisposing individuals to an increased risk of fracture. It represents a major public health concern globally, particularly among postmenopausal women, due to its association with significant morbidity, mortality, and reduced quality of life (1, 2). The decline in estrogen levels following menopause accelerates bone remodeling, leading to an imbalance between bone resorption and formation, which is the primary pathophysiological mechanism underlying PMO (3–5). Consequently, postmenopausal women are disproportionately affected, with a substantial proportion experiencing fragility fractures, especially of the hip, spine, and wrist (6, 7).

The pathogenesis of PMO is a complex, multi-factorial process primarily initiated by the cessation of ovarian function and the subsequent sharp decline in circulating estrogen levels (8, 9). Estrogen exerts a protective effect on bone mass through several key mechanisms. Its deficiency leads to a cascade of cellular events that tip the balance in favor of bone resorption (4, 10). At the cellular level, the loss of estrogen’s inhibitory action on the RANKL/RANK/OPG (osteoprotegerin) pathway is paramount. Estrogen deficiency causes an upregulation of RANKL (Receptor Activator of Nuclear Factor Kappa-B Ligand) production by osteocytes and bone marrow stromal cells (11, 12). RANKL binds to its receptor RANK on pre-osteoclasts, promoting their differentiation, activation, and survival, thereby dramatically increasing bone resorption. Concurrently, estrogen deficiency is associated with a decrease in OPG, a decoy receptor for RANKL, further tilting the RANKL/OPG ratio in favor of enhanced osteoclastic activity (9, 11). Beyond this central pathway, estrogen deficiency also increases the production of pro-inflammatory cytokines such as interleukin-1 (IL-1), interleukin-6 (IL-6), and tumor necrosis factor-alpha (TNF-α), which synergistically stimulate osteoclast formation and function (13, 14). Furthermore, estrogen plays a role in promoting apoptosis of osteoclasts and inhibiting apoptosis of osteoblasts (4, 10). Its loss therefore prolongs the lifespan of osteoclasts while shortening that of osteoblasts, leading to a net loss of bone. This uncoupling of bone remodeling results in a phase of rapid bone loss, particularly from trabecular bone, which has a higher surface area and turnover rate, explaining the early and pronounced occurrence of vertebral fractures in postmenopausal women (3, 8). This accelerated bone loss is superimposed on age-related changes, such as decreased intestinal calcium absorption, secondary hyperparathyroidism, and reduced osteoblast function, collectively contributing to the development of osteoporosis (15, 16) **Figure 1A**.

**Figure 1:**
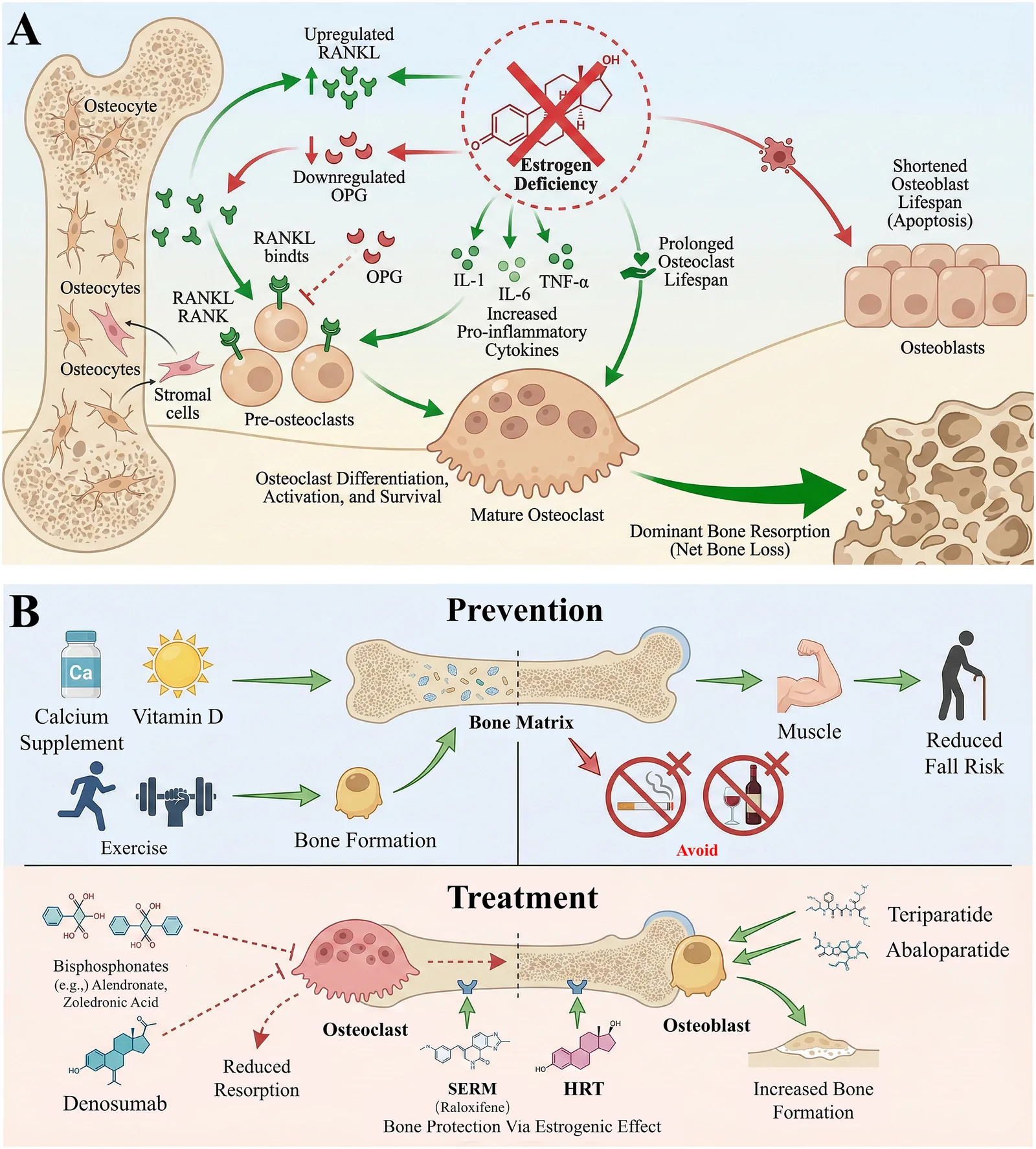
Pathogenesis and management of postmenopausal osteoporosis. **(A)** Pathogenesis: estrogen deficiency increases production of RANKL and inflammatory cytokines (IL-1, IL-6, TNF-α), while decreasing OPG. This promotes osteoclast differentiation, activation, and survival, leading to accelerated bone resorption. The lifespan of osteoblasts is simultaneously shortened, resulting in a net loss of bone mass, particularly from trabecular bone. **(B)** prevention and treatment: Preventive strategies focus on foundational measures: adequate intake of calcium and vitamin D, regular weight-bearing exercise, and smoking/alcohol restriction. For women at high risk, pharmacological interventions are employed. Antiresorptive agents (e.g., bisphosphonates, denosumab) inhibit osteoclast activity, while anabolic agents (e.g., teriparatide, abaloparatide) stimulate new bone formation. SERMs (e.g., raloxifene) and HRT provide targeted estrogenic effects.

In response to this significant health burden, a range of prevention and treatment strategies has been developed. Current mainstream approaches to managing osteoporosis in postmenopausal women encompass both non-pharmacological and pharmacological interventions (17, 18). Non-pharmacological strategies are foundational and include ensuring adequate intake of calcium and vitamin D, which are essential for bone mineralization (15, 19), alongside engaging in regular weight-bearing and muscle-strengthening exercises to stimulate bone formation and maintain muscle mass, thereby reducing fall risk (20). Lifestyle modifications, such as smoking cessation and limiting alcohol consumption, are also strongly recommended (21, 22). Pharmacological therapy is indicated for women at high risk of fracture. First-line treatments typically include antiresorptive agents, such as bisphosphonates (e.g., alendronate, zoledronic acid) and the RANKL inhibitor denosumab, which work by inhibiting osteoclast-mediated bone resorption (23, 24). For women at very high risk, anabolic agents like teriparatide (a recombinant parathyroid hormone) and abaloparatide, which directly stimulate new bone formation, are available (25, 26). Additionally, selective estrogen receptor modulators (SERMs), such as raloxifene, offer a tissue-specific estrogenic effect on bone (27). Hormone replacement therapy (HRT) was once a primary intervention but is now generally reserved for the management of menopausal symptoms in younger postmenopausal women at significant risk of osteoporosis, due to its associated risks (28) **Figure 1B**.

Despite the availability of these effective interventions, osteoporosis remains vastly underdiagnosed and undertreated, often only coming to clinical attention after the first fragility fracture occurs (22, 29). This “treatment gap” underscores the critical importance of early identification of individuals at high risk. While the central role of estrogen deficiency is undisputed, the individual risk of developing clinical osteoporosis is modulated by a complex interplay of various demographic, reproductive, genetic, lifestyle, and clinical factors (2, 30). A comprehensive understanding of these independent risk factors is crucial for developing accurate risk prediction models, enabling targeted screening programs, and implementing timely, individualized prevention strategies (31, 32).

Numerous observational studies have investigated the association between potential risk factors and PMO. However, the results have been inconsistent, with some studies reporting significant associations for certain factors while others do not. These discrepancies may stem from variations in study design, sample size, population characteristics, and statistical methodologies (1, 30, 33). Furthermore, many individual studies lack sufficient statistical power to detect modest but clinically important associations, particularly when examining multiple variables simultaneously using multivariate analysis (32).

To address these limitations and provide a more robust synthesis of the existing evidence, we conducted a systematic review and meta-analysis of observational studies that employed multivariate logistic regression analysis. This methodological approach is critical as it allows for the adjustment of potential confounders, thereby providing more reliable estimates of the independent effect of each risk factor (34, 35).The primary objectives of this study were to determine the pooled prevalence of osteoporosis in postmenopausal women and to quantitatively summarize the evidence regarding its independent risk factors based on adjusted effect estimates. This comprehensive analysis aims to inform clinical practice and public health policy by highlighting key modifiable and non-modifiable factors that contribute to osteoporosis risk in this vulnerable population.

## 2 Materials and methods

### 2.1 Information sources and literature search

This systematic review and meta-analysis adhered to the Preferred Reporting Items for Systematic Reviews and Meta-Analyses (PRISMA) guidelines and referenced the Meta-analysis of Observational Studies in Epidemiology (MOOSE) guidelines to ensure standardized and transparent reporting (36, 37). The study protocol was prospectively registered in the International Prospective Register of Systematic Reviews (PROSPERO: CRD420251274575). As all analyses utilized data from previously published studies, ethical approval was not required.

Two researchers (Zhao Qin, and Jun Liao) independently performed a comprehensive systematic search across eight bibliographic databases to ensure global coverage and reduce language bias. International databases comprised PubMed, Cochrane Library, Embase, and WoS; regional databases comprised CNKI, Wanfang Data, VIP Chinese Journal Database, and Sinomed. The search was conducted on 20 May 2026 with no lower date limit. The strategy was built on the PICO framework (Participants: postmenopausal women; Intervention/Exposure: candidate risk factors; Comparison: women without osteoporosis; Outcomes: osteoporosis diagnosis by BMD/T-score) (38). For each database we combined subject headings (e.g., MeSH in PubMed, Emtree-equivalent concepts in CNKI/Wanfang) with free-text keywords. English terms included “postmenopausal women”, “osteoporosis”, “risk factors”, “prevalence”; Chinese terms included “绝经”, “绝经后”, “骨质疏松”, “危险因素”, “患病率”. The complete search strings for every database are reproduced in **Supplementary Tables S1** and **S2**. In addition to database searching, the reference lists of all included studies and of published systematic reviews on PMO risk factors were manually screened to retrieve potentially missed records.

### 2.2 Inclusion and exclusion criteria

Inclusion criteria: a. Studies investigating the development of PMO; b. Observational studies employing multivariate logistic regression to assess risk factors for osteoporosis in this population; c. Officially published journal articles; d. Observational study designs (case-control, cohort, or cross-sectional studies); e. Studies providing sufficient data for the statistical analysis of two or more outcomes.

Exclusion criteria: a. Duplicate publications, conference abstracts, reviews, meta-analyses, case reports, studies with unrelated topics, or studies lacking detailed data; b. Studies using methods other than multivariate logistic regression to evaluate risk factors for PMO; c. Studies involving participants with conditions significantly affecting bone metabolism (e.g., malignancy, thyroid dysfunction, liver cirrhosis, rheumatoid arthritis); d. Low-quality studies.

### 2.3 Quality assessment and data extraction

Two reviewers independently assessed study quality. The Newcastle-Ottawa Scale (NOS) was used for case-control and cohort studies, with scores ranging from 0 to 9. Studies scoring 0–4 were considered low quality, 5–6 moderate quality, and 7–9 high quality. Only studies with an NOS score ≥ 7 were included (39). Cross-sectional studies were evaluated using the scale recommended by the Agency for Healthcare Research and Quality (AHRQ), consisting of 11 items rated as “yes”, “no”, or “unclear”. “yes” responses scored 1 point, while “no” or “unclear” scored 0, resulting in a total score of 0–11. Scores of 0–3 indicated low quality, 4–7 moderate quality, and 8–11 high quality (40). Disagreements between reviewers regarding risk of bias were resolved through discussion or by consulting a third reviewer.

Extracted data included the first author’s name, publication year, sample size, study design, and risk factors. The primary risk factors assessed were: Age, Age at menopause, Postmenopausal duration, Postmenopausal duration > 10 years, Number of births, With ≥ 2 births, Smoking, Lack of exercise, BMI, History of previous fracture, Diabetes mellitus, Estrogen replacement therapy, Family history of osteoporosis, Regular exercise, Hypertension, and Calcium supplementation. Basic study information was extracted independently by two reviewers. A risk factor was included in the analysis only if it was reported in three or more studies.

### 2.4 Statistical analysis and outcome measures

Statistical analysis was performed using STATA 15.0 software. Dichotomous data were analyzed using pooled OR and their 95% CI as effect measures. Heterogeneity among included studies was assessed using the Q test and I² statistic. A fixed-effects model was employed for meta-analysis if the P-value from the Q test was ≥ 0.1 and the I² value was ≤ 50%, indicating no significant statistical heterogeneity. A random-effects model was used if the P-value was < 0.1 or I² > 50%, indicating significant heterogeneity. Sensitivity analyses for risk factors were conducted by altering the effect model and using the “leave-one-out” method.

## 3 Results

### 3.1 Overview of literature search

Based on the predefined search strategy, 8,388 potentially relevant records were initially identified. After removing duplicates, 6,061 records underwent initial screening of titles and abstracts. Subsequently, 5,967 records were excluded for clearly not meeting the inclusion criteria. Full-text assessment was performed for 94 articles. Following rigorous quality assessment and data extraction, a total of 37 studies (5, 33, 41–75) were finally included in this systematic review and meta-analysis. The literature screening flowchart is shown in **Figure 2**.

**Figure 2:**
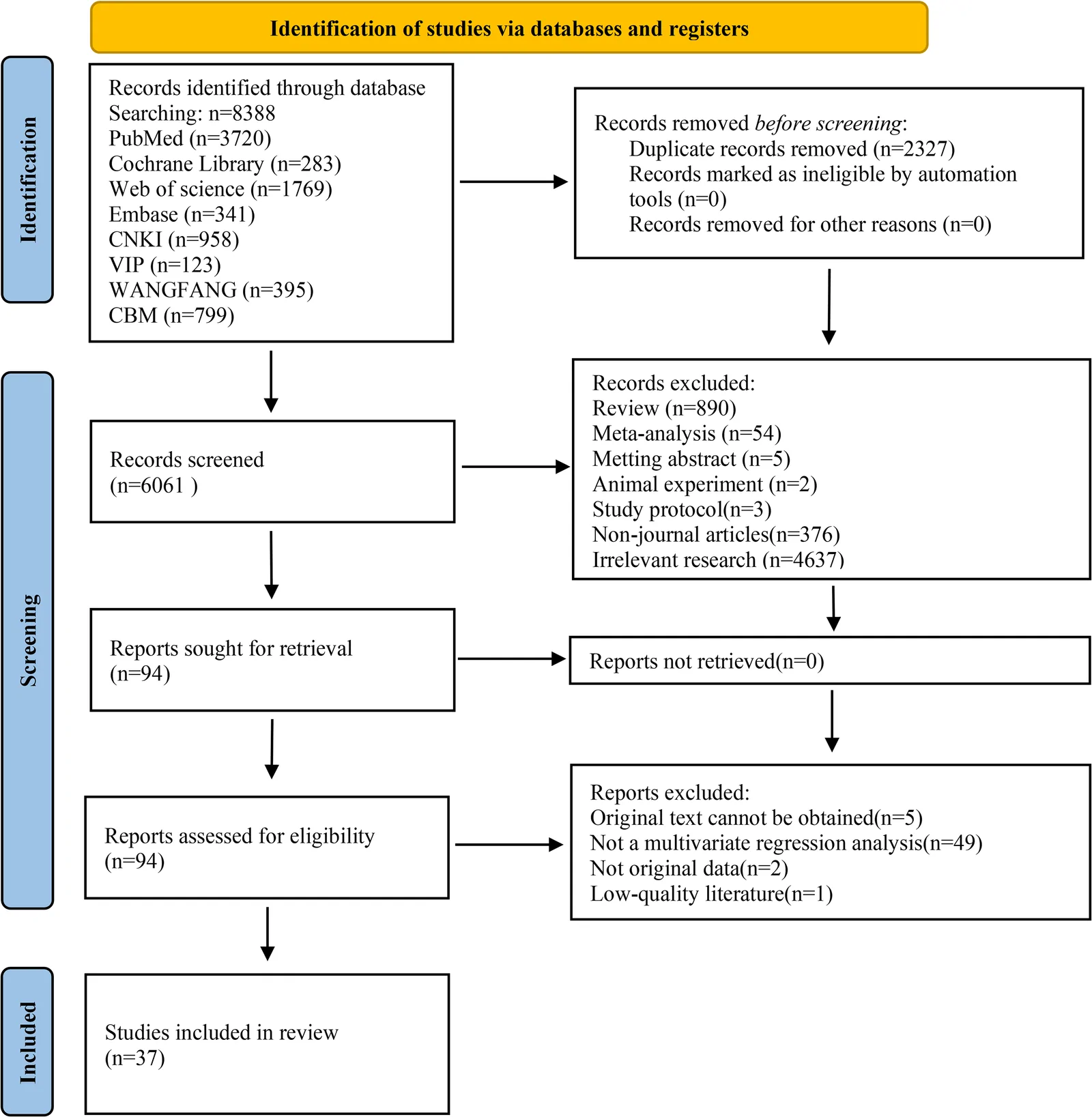
PRISMA flow diagram of the study selection process. A total of 8388 records were identified from 8 databases. After removal of 2327 duplicates, 6061 unique records were screened. Based on titles and abstracts, 5967 records were excluded (review (n=890), meta-analysis (n=54), metting abstract (n=5), animal experiment (n=2), study protocol (n=3), non-journal articles (n=376), irrelevant research (n=4637)), leaving 94 potentially eligible reports. Full-text assessment of these 94 reports was performed. A total of 57 reports were excluded [Original text cannot be obtained (n=5), not a multivariate regression analysis (n=49), not original data(n=2), low-quality literature (n=1)]. Ultimately, 37 studies were included in the meta-analysis.

### 3.2 Characteristics of included studies

A total of 37 studies were included, encompassing 32,366 participants. Among these, 36 studies (involving 23,722 participants, with 8,025 diagnosed with PMO) reported a pooled prevalence of osteoporosis of 33.87%. The included studies originated from multiple countries: China (n = 27) (49–75) (all Chinese publications); Korea (n=1) (41) (Korean publication); Turkey (n=3) (33, 45, 46); Argentina (n=1) (42); Iran (n=1) (43); India (n = 1) (5); Belgrade (n = 1) (44); Japan (n = 1) (47); and Nepal (n = 1) (48). The publication dates ranged from 2005 to 2025. The included study designs comprised cross-sectional studies (n = 12), case-control studies (n = 22), and cohort studies (n = 3). The average score for cross-sectional studies using the AHRQ tool was 8.16; the average score for case-control and cohort studies using the NOS was 7.72, indicating all included studies were of high quality (**Table 1**).

**Table 1:** Characteristics of included literature studies.

Author	Year	Country	Study design	Sample size			Age		NOS/AHRQ
				Total	Osteoporotics	No-Osteoporotics	Osteoporotics	No- Osteoporotics	
Jang et al. (41)	2006	Korea	CSS	362	182	180	70.6 ± 8.2	65.5 ± 8.6	8
Masoni et al. (42)	2005	Argentina	CCS	86	53	33	50.3 ± 1.0	55.6 ± 1.3	8
Akkus et al. (33)	2005	Turkey	CCS	351	225	126	62.38 ± 8.21	59.38 ± 7.67	7
Matin et al. (43)	2015	Iran	CS	8644	NR	NR	NR	NR	9
John et al. (5)	2024	India	CCS	383	165	218	NR	NR	8
Grgurevic et al. (44)	2010	Belgrade	CCS	200	100	100	64 ± 9.01	64 ± 9.01	9
Sahin et al. (45)	2015	Turkey	CCS	537	239	298	58.1 ± 6.4	63.0 ± 7.8	9
Taskale et al. (46)	2010	Turkey	CSS	1100	349	751	61.5 ± 8.6	55.3 ± 8.1	8
Miura et al. (47)	2008	Japan	CSS	339	67	292	NR	NR	8
Pandey et al. (48)	2024	Nepal	CSS	384	82	302	67.52 ± 8.84	55.70 ± 7.69	8
Wang et al. (49)	2009	China	CCS	412	129	283	46–71	45–73	8
Ruan et al. (50)	2013	China	CCS	912	313	599	NR	NR	8
Zhao et al. (51)	2013	China	CCS	80	44	36	NR	NR	7
Hu et al. (52)	2015	China	CCS	200	90	110	NR	NR	8
Li et al. (53)	2016	China	CCS	819	354	465	65.37 ± 8.56	59.54 ± 6.70	7
Weng et al. (54)	2017	China	CCS	148	148	148	NR	NR	7
Qin et al. (55)	2017	China	CSS	123	84	39	67.64 ± 6.28	56.74 ± 5. 3	8
Xu et al. (56)	2018	China	CCS	147	62	85	64.91 ± 7.88	60.89 ± 6.5	7
Yang et al. (57)	2020	China	CSS	537	193	344	71.83 ± 5.42	67.38 ± 5.14	8
Zhang et al. (58)	2020	China	CCS	150	36	114	NR	NR	7
Gu et al. (59)	2020	China	CSS	1562	535	1027	68.13 ± 5.48	61.42 ± 6.35	8
Xu et al. (60)	2020	China	CSS	309	200	109	NR	NR	8
Jiang et al. (61)	2021	China	CSS	580	246	334	NR	NR	8
Zhao et al. (62)	2021	China	CSS	6258	811	5447	55–65	55–65	
Wang et al. (63)	2021	China	CSS	1776	869	907	NR	NR	8
Zhao et al. (64)	2022	China	CCS	200	95	105	68.22 ± 2.12	68.22 ± 2.12	7
Tian et al. (65)	2024	China	CCS	540	180	360	54.80 ± 5.30	54.24 ± 5.18	8
Cheng et al. (66)	2024	China	CCS	569	306	263	51–80	51–80	7
Wang et al. (67)	2023	China	CCS	323	185	138	58.96 ± 4.28	58.67 ± 3.93	8
Feng et al. (68)	2022	China	CCS	600	240	360	51–71	54–78	7
Wu et al. (69)	2022	China	CCS	420	174	246	42–80	42–80	7
Lin et al. (70)	2022	China	CSS	700	214	486	NR	NR	8
Qu et al. (71)	2022	China	CCS	215	88	127	63.58 ± 5.41	63.58 ± 5.41	8
Wang et al. (72)	2023	China	CS	319	159	160	67.2 ± 6.4	65.3 ± 6.3	9
Liu et al. (73)	2025	China	CS	676	183	493	NR	NR	9
Li et al. (74)	2025	China	CCS	198	98	100	60.35 ± 0.49	60.25 ± 0.52	7
You et al. (75)	2009	China	CCS	500	250	250	62.8 ± 8.5	52.6 ± 4.2	7

CSS, Cross-sectional study; CCS, Case-Control Study; CS, Cohort study; NOS, Newcastle–Ottawa Scale; AHRQ, Agency of Healthcare Research and Quality; NR, Not reported.

### 3.3 Age

13 studies (5, 33, 43, 47, 48, 50, 52–54, 62, 66, 67, 73) analyzed the impact of age on PMO. Heterogeneity testing revealed high heterogeneity among studies (I² = 92.8%, P < 0.001); thus, a random-effects model was used. The pooled result showed an OR of 1.16 (95% CI: 1.11–1.22, P < 0.001) (**Figure 3A**; **Table 2**). This indicates that for each one-year increase in age, the risk of PMO increases by an average of 16%. This is primarily related to age-related mechanisms of bone loss, including declining osteoblast function, reduced bone formation, and accompanying vitamin D deficiency and secondary hyperparathyroidism with advancing age. This suggests that even after accounting for menopause factors, aging itself is always associated with increased PMO risk.

**Figure 3:**
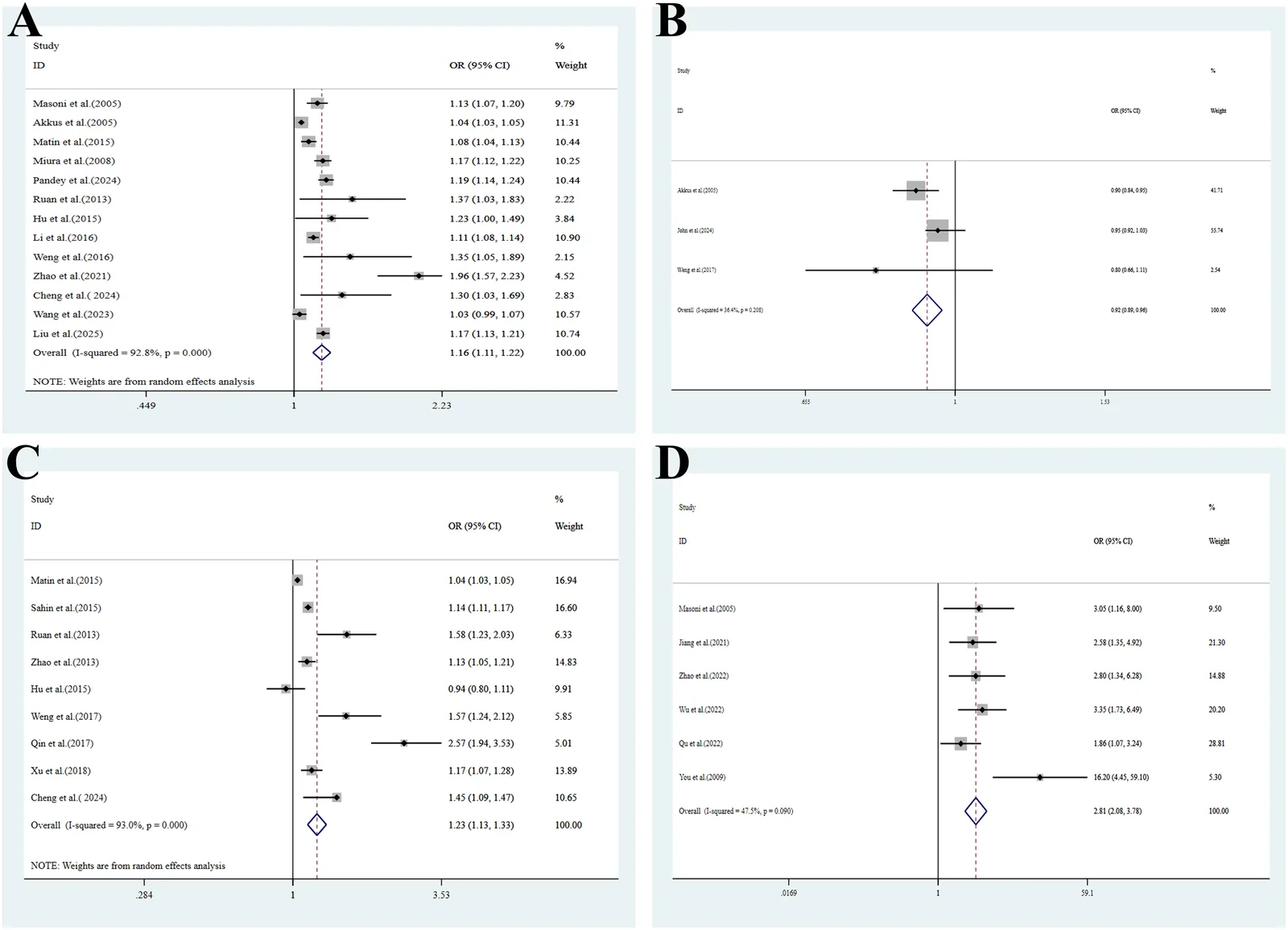
Forest plots of meta-analysis for menopausal timing and duration-related risk factors associated with osteoporosis in postmenopausal women. **(A)** Age: The overall pooled OR from random-effects meta-analysis was 1.16 (95% CI: 1.11–1.22, p < 0.001), indicating a significant association between increasing age and higher odds of osteoporosis. Heterogeneity was high (I² = 92.8%, p < 0.001). **(B)** Age at menopause: The pooled OR was 0.92 (95% CI: 0.89–0.96, p < 0.001), suggesting that older age at menopause is a significant risk factor. Heterogeneity was very high (I² = 36.4%, p = 0.208). **(C)** Postmenopausal duration: The pooled OR was 1.23 (95% CI: 1.13–1.33, p < 0.001), indicating a significant association between longer postmenopausal duration and increased odds of osteoporosis. Heterogeneity was very high (I² = 93.0%, p < 0.001). **(D)** Postmenopausal duration >10 years: The pooled OR was 2.81 (95% CI: 2.08–3.78, p < 0.001), demonstrating that more than 10 years postmenopause is strongly associated with osteoporosis. Heterogeneity was moderate (I² = 47.5%, p = 0.090). For each subplot, individual study results are shown as ORs with 95% confidence intervals. The size of each square corresponds to the study’s weight in the random-effects model. The diamond represents the pooled OR and 95% CI. The vertical dashed line indicates the line of no effect (OR = 1).

**Table 2:** Detailed data on 16 inclusion factors for the osteoporosis of postmenopausal women.

Inclusion factors	Literatures(n)	Heterogeneity test		Effect model	OR (95%CI)	P-value	Reference
		I^2^%	P-value				
**Age**	13	92.8	<0.001	Random effects	1.16(1.11, 1.22)	<0.001*	(5, 33, 43, 47, 48, 50, 52–54, 62, 66, 67, 73)
**Age at menopause**	3	36.4	0.208	Fixed effects	0.92(0.89, 0.96)	<0.001*	(5, 33, 54)
**Postmenopausal duration**	9	93	<0.001	Random effects	1.23(1.13, 1.33)	<0.001*	(43, 45, 50–52, 54–56, 66)
**Postmenopausal duration >10 years**	6	47.5	0.09	Fixed effects	2.81(2.08, 3.78)	<0.001*	(42, 61, 64, 69, 71, 75)
**Number of births**	8	71.8	0.001	Random effects	1.32(1.03, 1.69)	0.026*	(50, 53, 54, 56, 57, 59, 66, 70)
**With ≥2 births**	3	0	0.594	Fixed effects	3.07(2.10, 4.50)	<0.001*	(61, 64, 71)
Family history of osteoporosis	5	88.2	<0.001	Random effects	1.60(0.86, 2.97)	0.139	(57, 59, 67, 68, 70)
**Smoking**	4	26.8	0.251	Fixed effects	1.55(1.24, 1.93)	<0.001*	(48, 53, 57, 63)
Regular exercise	8	91.30	<0.001	Random effects	0.81(0.50, 1.31)	0.383	(41, 43, 46, 54, 56, 63, 64, 66)
**Lack of exercise**	6	83.50	<0.001	Random effects	1.66(1.07, 2.56)	0.023*	(53, 57, 58, 61, 67, 71)
**BMI**	11	85.2	<0.001	Random effects	0.80(0.75, 087)	<0.001*	(5, 43, 45, 48, 52–57, 60)
**History of previous fracture**	9	88.2	<0.001	Random effects	1.86(1.22, 2.85)	0.004*	(42, 44, 49, 57, 63, 67, 69, 70, 75)
**Diabetes mellitus**	10	92.6	<0.001	Random effects	2.24(1.38, 3.64)	0.001*	(43, 57, 59, 61, 65, 67–69, 71, 75)
Hypertension	3	89.2	<0.001	Random effects	1.25(0.62, 2.56)	0.532	(57, 59, 68)
**Estrogen replacement therapy**	5	90.4	<0.001	Random effects	0.45(0.23, 0.90)	0.024*	(61, 64, 69, 70, 74)
Calcium supplementation	8	94.7	<0.001	Random effects	0.77(0.35, 1.69)	0.508	(61, 64, 65, 67, 70, 71, 74, 75)

*indicates P< 0.05, indicating that this factor is a risk factor for Osteoporosis of postmenopausal women.

Bold values denote risk factors or protective factors.

Given the high heterogeneity (I² = 92.8%, P < 0.001), we further explored its origins beyond random chance. A re-evaluation of the primary studies revealed that this variability was largely driven by fundamental discrepancies in how age was reported across cohorts—some studies provided mean ± SD, while others applied varying categorized thresholds without reporting exact numerical values, precluding granular subgroup comparisons. Consequently, we performed subgroup analysis stratified by geographic region (**Supplementary Figure S1A**), which partially mitigated the heterogeneity (I² decreased to 86.4% in East Asian subgroups), though it remained elevated.

To complement this, we inspected the funnel plot (**Supplementary Figure S2A**), which showed that the effect estimates were roughly symmetrically distributed around the pooled effect line without significant unilateral missing, suggesting a low likelihood of substantial publication bias. This visual impression was further evaluated using Begg’s and Egger’s tests (76) (**Supplementary Table S3**): the Begg’s test yielded a non-significant result (p = 0.714), whereas the Egger’s test was significant (p = 0.003). This dissociation between the two tests—together with the largely symmetric funnel plot—suggests that the observed asymmetry was attributable to methodological diversity in age reporting (e.g., inconsistent reporting units and categorized thresholds) rather than pure publication bias.

Sensitivity analysis (leave-one-out method) confirmed that excluding any single study did not alter the direction of the pooled effect estimate or its statistical significance (**Supplementary Figure S3A**). Therefore, despite the persistent statistical heterogeneity rooted in the structural limitations of age data reporting, the integrated analysis based on current evidence demonstrates reliable stability.

### 3.4 Age at menopause

3 studies (5, 33, 54) investigated the effect of age at menopause. Heterogeneity was low (I² = 36.4%, P = 0.208), leading to a fixed-effects model. The pooled OR was 0.92 (95% CI: 0.89–0.96, P < 0.001) (**Figure 3B**; **Table 2**). This indicates that each one-year delay in menopausal age is associated with an 8% reduction in PMO risk. Later menopause implies a longer lifetime exposure to the protective effects of endogenous estrogen, which is associated with higher peak bone mass accumulation and a delayed onset of rapid bone loss. This serves as an important protective indicator for assessing an individual’s long-term bone health. We note that this conclusion is derived from three included studies; while informative, it should be interpreted with caution when considering clinical implications.

### 3.5 Postmenopausal duration

9 studies (43, 45, 50–52, 54–56, 66) analyzed the effect of postmenopausal duration. High heterogeneity was present (I² = 93%, P < 0.001), leading to a random-effects model. The pooled OR was 1.23 (95% CI: 1.13–1.33, P < 0.001) (**Figure 3C**; **Table 2**). This indicates that for each additional year post-menopause, the risk of PMO increases by 23%. This directly reflects the cumulative effect and duration of the estrogen-deficient state. The early postmenopausal years (5–10 years) are characterized by a phase of rapid bone loss with high bone turnover rates, primarily affecting trabecular bone. Longer duration is associated with more severe deterioration of bone microarchitecture and higher fracture risk.

For postmenopausal duration, substantial heterogeneity was also observed (I² = 93%, P < 0.001). Inconsistent reporting formats limited granular meta-regression; therefore, we performed subgroup analysis stratified by geographic region. This approach partially reduced the variability (I² decreased to 83.8% in East Asia; **Supplementary Figure S1B**), suggesting that regional differences in menopausal age recall and data collection likely contributed to, but did not fully account for, the observed heterogeneity.

We then assessed potential small-study effects. The funnel plot appeared largely symmetric (**Supplementary Figure S2B**), arguing against substantial publication bias. This visual impression was supported by a non-significant Begg’s test (p = 0.211), whereas the Egger’s test was significant (p = 0.01; **Supplementary Table S3**). This dissociation may reflect methodological diversity in defining and reporting menopausal duration rather than selective publication.

Finally, leave-one-out sensitivity analysis suggested the robustness of the pooled estimate (**Supplementary Figure S3B**). Therefore, despite persistent heterogeneity likely rooted in the structural limitations of menopausal duration data, the integrated analysis provides a stable estimate based on currently available evidence.

### 3.6 Postmenopausal duration >10 years

6 studies (42, 61, 64, 69, 71, 75) evaluated this factor. Heterogeneity was acceptable (I² = 47.5%, P = 0.09), leading to a fixed-effects model. The pooled OR was 2.81 (95% CI: 2.08–3.78, P < 0.001) (**Figure 3D**; **Table 2**). This indicates that women who have been postmenopausal for more than 10 years have a 2.81-fold higher risk of PMO compared to those within 10 years. This marks a critical clinical time point, suggesting that patients may have passed the phase of rapid bone loss and entered a stage characterized by cortical bone loss and continued slow bone mineral density decline, with significantly increased fracture risk. It serves as an important indicator for intensive screening and intervention.

### 3.7 Number of births

8 studies (50, 53, 54, 56, 57, 59, 66, 70) analyzed the effect of the number of births. Moderate heterogeneity was present (I² = 71.8%, P = 0.001), leading to a random-effects model. The pooled OR was 1.32 (95% CI: 1.03–1.69, P = 0.026) (**Figure 4A**; **Table 2**). This suggests that multiple births (e.g., more than two) may be a weak risk factor for PMO. Potential mechanisms include the substantial transfer of maternal calcium to the fetus or infant during pregnancy and lactation, which may lead to reversible or irreversible bone loss if nutritional replenishment is insufficient. Furthermore, multiple births may also correlate with lower socioeconomic status and nutritional status.

**Figure 4:**
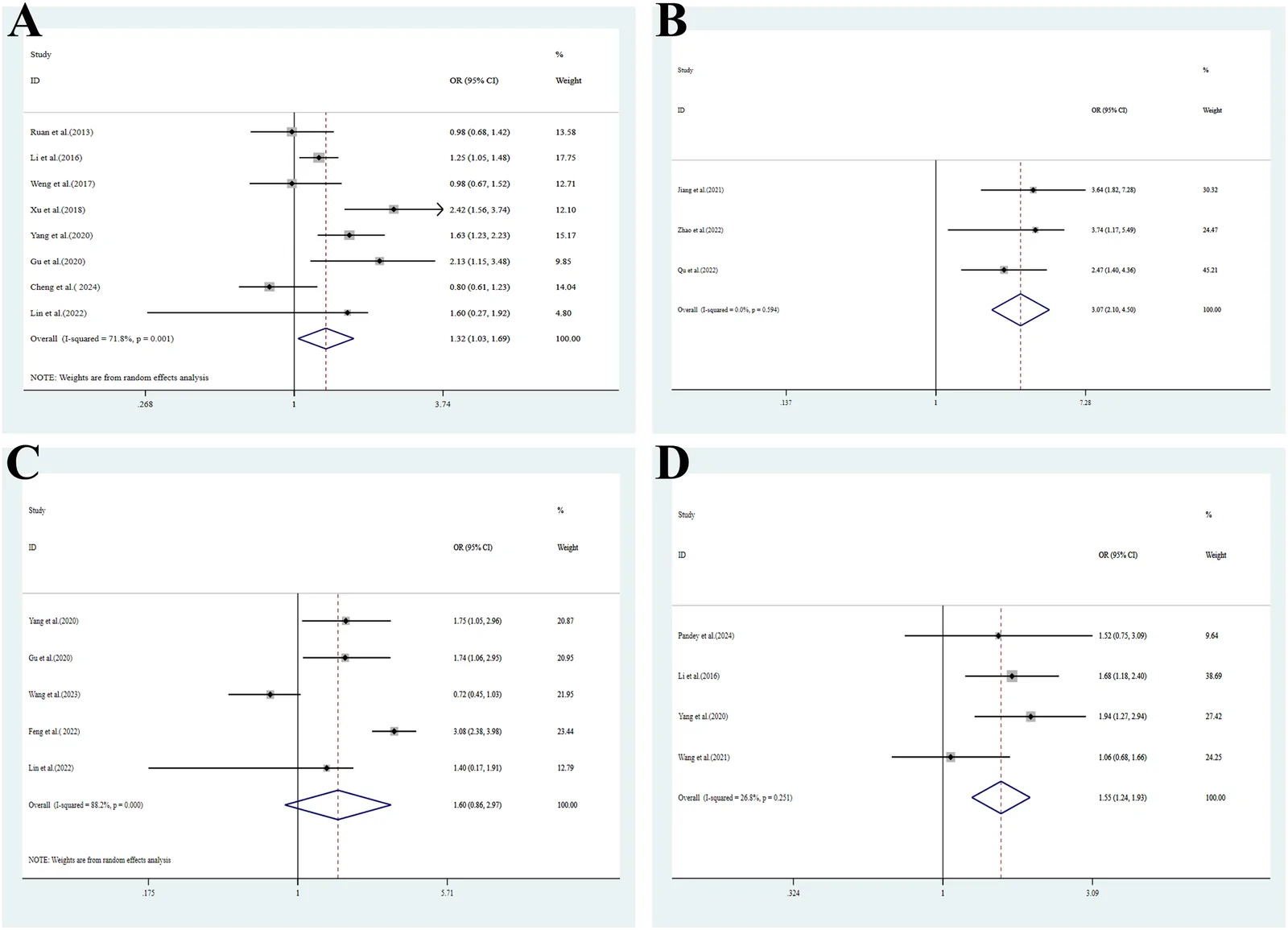
Forest plots of meta-analysis for reproductive, familial, and lifestyle-related risk factors associated with osteoporosis in postmenopausal women. **(A)** Number of births: The overall pooled OR from random-effects meta-analysis was 1.32 (95% CI: 1.03–1.69, p = 0.026), indicating a significant association between parity and increased odds of osteoporosis. Heterogeneity was high (I²= 71.8%, p= 0.001). **(B)** With ≥2 births: The pooled OR for having two or more births was 3.07 (95% CI: 2.10–4.50, p < 0.001), suggesting multiparity is a significant risk factor. Heterogeneity was negligible (I²= 0, p= 0.594). **(C)** Family history of osteoporosis: The pooled OR was 1.60 (95% CI: 0.86–2.97, p = 0.139), indicating no statistically significant association in the overall analysis Heterogeneity was very high (I²= 88.2%, p< 0.001). **(D)** Smoking: the pooled OR was 1.55 (95% CI: 1.24–1.93, p < 0.05), confirming smoking as a significant risk factor. Heterogeneity was low (I²= 26.8%, p= 0.251).

For number of births, substantial heterogeneity was noted (I² = 71.8%, P = 0.001). Similar to age, inconsistent reporting formats across studies precluded granular meta-regression. Notably, all included studies examining this factor were derived exclusively from Chinese cohorts, rendering subgroup analysis by geographic region unfeasible.

Assessment of small-study effects revealed that the funnel plot was largely symmetric (**Supplementary Figure S2C**). This visual impression was corroborated by both non-significant Begg’s (p = 0.322) and Egger’s tests (p = 0.611; **Supplementary Table S3**), arguing against substantial publication bias or methodological asymmetry.

Finally, leave-one-out sensitivity analysis suggested the robustness of the pooled estimate (**Supplementary Figure S3C**). Therefore, despite persistent statistical heterogeneity likely rooted in unmeasured clinical or genetic factors within this specific population, the integrated analysis provides a stable estimate based on currently available evidence.

### 3.8 With ≥2 births

3 studies (61, 64, 71) specifically assessed the effect of having ≥ 2 births. No heterogeneity was observed among studies (I² = 0, P = 0.594), leading to a fixed-effects model. The pooled OR was 3.07 (95% CI: 2.10–4.50, P < 0.001) (**Figure 4B**; **Table 2**). This represents a very strong risk signal. Compared to “number of births” as a continuous variable, this categorical variable (≥ 2 births) more clearly defines a high-risk threshold. It suggests that in clinical history-taking, “having ≥ 2 births” should be inquired about and evaluated as an important reproductive history risk factor. Although the effect size is substantial, it is important to acknowledge that this conclusion rests on just three included studies; thus, direct translation into clinical screening protocols should be approached with caution pending further validation.

### 3.9 Family history of osteoporosis

5 studies (57, 59, 67, 68, 70) investigated the effect of family history. High heterogeneity was present (I² = 88.2%, P < 0.001), leading to a random-effects model. The pooled OR was 1.60 (95% CI: 0.86–2.97, P = 0.139) (**Figure 4C**; **Table 2**). This indicates that although the point estimate (OR = 1.60) suggests a trend towards increased risk, the wide confidence interval includes 1, rendering the result statistically non-significant. This may be due to inaccurate reporting of family history or differing definitions across studies. While the role of genetic factors (e.g., vitamin D receptor gene polymorphisms) in osteoporosis is established, this meta-analysis could not confirm a strong independent association, potentially requiring larger samples or more precise genetic measurements for validation.

For family history of osteoporosis, substantial heterogeneity was also observed (I² = 88.2%, P < 0.001). The binary nature of this outcome (“yes/no”) without further stratification (e.g., age of onset in relatives) limited our ability to explore clinical subgroups. Consistent with the findings for number of births, all included studies were derived exclusively from Chinese cohorts, precluding subgroup analysis by geographic region.

Assessment of small-study effects demonstrated a largely symmetric funnel plot (**Supplementary Figure S2D**). This visual impression was corroborated by both non-significant Begg’s (p = 0.624) and Egger’s tests (p = 0.384; **Supplementary Table S3**), arguing against substantial publication bias or methodological asymmetry.

Finally, leave-one-out sensitivity analysis suggested the robustness of the pooled estimate (**Supplementary Figure S3D**). Therefore, despite persistent statistical heterogeneity likely rooted in unmeasured genetic or phenotypic variations within this specific population, the integrated analysis provides a stable estimate based on currently available evidence.

### 3.10 Smoking

4 studies (48, 53, 57, 63) analyzed the impact of smoking. Heterogeneity was low (I² = 26.8%, P = 0.251), leading to a fixed-effects model. The pooled OR was 1.55 (95% CI: 1.24–1.93, P < 0.001) (**Figure 4D**; **Table 2**). This identifies smoking as a clear, modifiable risk factor. Toxic substances in tobacco (e.g., nicotine, cadmium) can directly inhibit osteoblast activity, interfere with calcium absorption, and may exert anti-estrogenic effects, leading to bone loss. Smoking cessation should be considered a core component of primary prevention for PMO.

For smoking exposure, definitions varied considerably across studies, ranging from binary “yes/no” classifications without specific thresholds to criteria based on established guidelines (e.g., WHO-defined passive smoking) or quantitative consumption metrics (77). This lack of standardized definitions likely introduced methodological diversity that could not be fully captured by meta-regression.

### 3.11 Regular exercise

8 studies (41, 43, 46, 54, 56, 63, 64, 66) evaluated the impact of regular exercise. High heterogeneity was present (I² = 91.3%, P < 0.001), leading to a random-effects model. The pooled OR was 0.81 (95% CI: 0.50–1.31, P = 0.383) (**Figure 5A**; **Table 2**). The point estimate suggests a protective trend, but the result is not significant. The high heterogeneity likely stems from significant variations across studies in defining “regular exercise” regarding type, intensity, and frequency. Although this analysis did not yield a significant conclusion, the benefits of weight-bearing and resistance exercise for bone health are well-established by substantial basic research and clinical practice, and their importance cannot be ignored.

**Figure 5:**
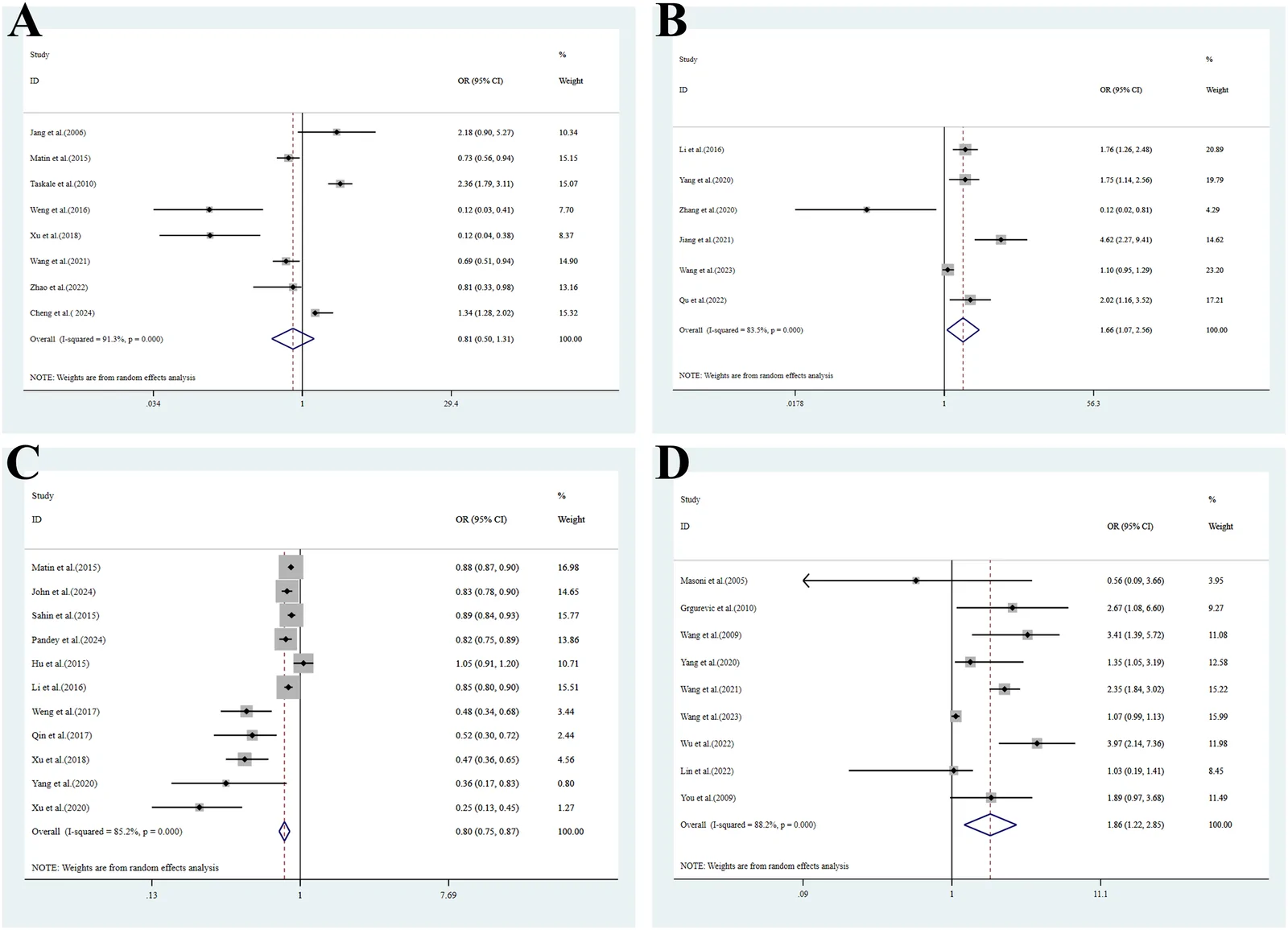
Forest plots of meta-analysis for lifestyle-related and clinical risk factors associated with osteoporosis in postmenopausal women. **(A)** Regular exercise: The overall pooled OR from random-effects meta-analysis was 0.81 (95% CI: 0.50–1.31, p = 0.383), suggesting a significant association between regular exercise and osteoporosis risk. Heterogeneity was high (I²= 91.3%, p< 0.001). **(B)** Lack of exercise: The pooled OR was 1.66 (95% CI: 1.07–2.56, p = 0.023), confirming lack of exercise as a significant risk factor. Heterogeneity was high (I²= 83.5%, p< 0.001). **(C)** BMI: The pooled OR was 0.80 (95% CI: 0.75–0.87, p< 0.001), indicating no statistically significant association in the overall analysis. Heterogeneity was moderate (I²= 85.2%, p< 0.001). **(D)** History of previous fracture: The pooled OR was 1.86 (95% CI: 1.22–2.85, p = 0.004), suggesting that prior fracture history is a significant risk factor. Heterogeneity was substantial (I²= 88.2%, p< 0.001).

For regular exercise, substantial heterogeneity was noted (I² = 91.3%, P < 0.001). In addition to the binary reporting format, definitional criteria varied widely across studies. While some applied explicit, quantifiable thresholds (e.g., frequency, duration, and intensity), the majority relied on binary self-reports via questionnaires without specific criteria. This heterogeneity in defining what constitutes “regular exercise” directly contributed to the observed statistical variability. Therefore, we performed subgroup analysis stratified by geographic region. Within the East Asian subgroup, the I² estimate was 91.5% (**Supplementary Figure S1C**). While this represents a marginal numerical increase from the overall estimate, it is consistent with the notion that the exclusion of studies from other regions removed potential sources of between-subgroup variability, thereby isolating and highlighting the substantial methodological and clinical heterogeneity intrinsic to East Asian populations (e.g., variations in self-reported activity assessment and population-specific lifestyle patterns). This suggests that regional differences alone do not fully explain the observed variance, and that unmeasured factors within East Asia likely play a dominant role.

We then assessed potential small-study effects. The funnel plot appeared largely symmetric (**Supplementary Figure S2E**), arguing against substantial publication bias. This visual impression was supported by a non-significant Begg’s test (p = 0.216) and Egger’s test (p = 0.27; **Supplementary Table S3**). This dissociation may reflect methodological diversity in defining and assessing physical activity rather than selective publication.

Finally, leave-one-out sensitivity analysis suggested the robustness of the pooled estimate (**Supplementary Figure S3E**). Therefore, despite persistent and unmitigated heterogeneity likely rooted in the structural limitations of self-reported exercise data, the integrated analysis provides a stable estimate based on currently available evidence.

### 3.12 Lack of exercise

6 studies (53, 57, 58, 61, 67, 71) analyzed the impact of lack of exercise. High heterogeneity was present (I² = 83.5%, P < 0.001), leading to a random-effects model. The pooled OR was 1.66 (95% CI: 1.07–2.56, P = 0.023) (**Figure 5B**; **Table 2**). Compared to the result for “regular exercise”, “lack of exercise” as a risk factor provides a clearer positive signal. This suggests that, relative to the uncertain benefit of “active exercise”, a “sedentary lifestyle” poses a more definite threat to bone health, as it implies a lack of necessary mechanical loading stimuli for bones, leading to weakened bone formation.

For lack of exercise, substantial heterogeneity was also observed (I² = 83.5%, P < 0.001). Beyond the limitations of binary “yes/no” reporting, operational definitions varied substantially across studies. Most studies relied on self-designed questionnaires without reporting specific thresholds for frequency, duration, or intensity, while a few adopted frequency-based criteria. This inconsistency in measurement tools and the absence of standardized definitions likely introduced additional clinical heterogeneity. Notably, all included studies examining this factor were derived exclusively from Chinese cohorts, precluding subgroup analysis by geographic region.

Assessment of small-study effects demonstrated a largely symmetric funnel plot (**Supplementary Figure S2F**). This visual impression was corroborated by both non-significant Begg’s (p = 0.573) and Egger’s tests (p = 0.401; **Supplementary Table S3**), arguing against substantial publication bias or methodological asymmetry.

Finally, leave-one-out sensitivity analysis suggested the robustness of the pooled estimate (**Supplementary Figure S3F**). Therefore, despite persistent statistical heterogeneity likely rooted in unmeasured lifestyle variations and self-reporting biases within this specific population, the integrated analysis provides a stable estimate based on currently available evidence.

### 3.13 BMI

11 studies (5, 43, 45, 48, 52–57, 60) investigated the impact of BMI. High heterogeneity was present (I² = 85.2%, P < 0.001), leading to a random-effects model. The pooled OR was 0.80 (95% CI: 0.75–0.87, P < 0.001) (**Figure 5C**; **Table 2**). This indicates that a higher BMI is a significant protective factor. Mechanisms include: a. Increased mechanical load stimulating bones; b. Aromatase in adipose tissue converting androstenedione to estrogen, providing an additional postmenopausal estrogen source; c. Higher body weight often correlates with higher nutrient intake. However, it should be noted that obesity may increase fall risk, so its overall impact on fractures requires comprehensive assessment.

For BMI, substantial heterogeneity was observed (I² = 85.2%, P < 0.001). Inconsistent reporting formats limited granular meta-regression; therefore, we performed subgroup analysis stratified by geographic region. This approach partially reduced the variability (I² decreased to 29.9% in West Asia; **Supplementary Figure S1D**), suggesting that regional differences in BMI classification standards and body composition assessment likely contributed to, but did not fully account for, the observed heterogeneity.

We then assessed potential small-study effects. The funnel plot suggested some asymmetry (**Supplementary Figure S2G**), which was statistically supported by both significant Begg’s (p = 0.024) and Egger’s tests (p = 0.011; **Supplementary Table S3**). While this raises the possibility of small-study effects, the asymmetry may reflect methodological diversity in BMI measurement and categorization across different populations rather than selective publication, given the well-documented variations in BMI thresholds between Western and Asian cohorts (78, 79).

Finally, leave-one-out sensitivity analysis suggested the robustness of the pooled estimate (**Supplementary Figure S3G**). Therefore, despite persistent heterogeneity likely rooted in the structural limitations of BMI data reporting and population-specific cutoffs, the integrated analysis provides a stable estimate based on currently available evidence.

### 3.14 History of previous fracture

9 studies (42, 44, 49, 57, 63, 67, 69, 70, 75) analyzed the impact of a history of previous fracture. High heterogeneity was present (I² = 88.2%, P < 0.001), leading to a random-effects model. The pooled OR was 1.86 (95% CI: 1.22–2.85, P = 0.004) (**Figure 5D**; **Table 2**). This is one of the strongest clinical predictors. Individuals with a history of low-trauma fracture (especially vertebral or hip) already have compromised bone quality and strength, placing them at very high risk for subsequent fractures, consistent with the “fracture begets fracture” principle. Such patients should unconditionally initiate intensive pharmacological therapy.

For history of previous fracture, substantial heterogeneity was also observed (I² = 88.2%, P < 0.001). The binary “yes/no” outcome lacked stratification regarding fracture site (e.g., vertebral vs. hip) or trauma severity, which limited our ability to explore clinical subgroups; therefore, we performed subgroup analysis stratified by geographic region. This approach partially reduced the variability (I² decreased to 79.0% in East Asia; **Supplementary Figure S1E**), suggesting that regional differences in fracture documentation standards and diagnostic practices likely contributed to, but did not fully account for, the observed heterogeneity.

We then assessed potential small-study effects. The funnel plot appeared largely symmetric (**Supplementary Figure S2H**), arguing against substantial publication bias. This visual impression was supported by a non-significant Begg’s test (p = 0.404) and Egger’s test (p = 0.065; **Supplementary Table S3**).

Finally, leave-one-out sensitivity analysis suggested the robustness of the pooled estimate (**Supplementary Figure S3H**). Therefore, despite persistent heterogeneity likely rooted in the structural limitations of binary fracture reporting and population-specific diagnostic criteria, the integrated analysis provides a stable estimate based on currently available evidence.

### 3.15 Diabetes mellitus

10 studies (43, 57, 59, 61, 65, 67–69, 71, 75) evaluated the impact of diabetes mellitus. High heterogeneity was present (I² = 92.6%, P < 0.001), leading to a random-effects model. The pooled OR was 2.24 (95% CI: 1.38–3.64, P = 0.001) (**Figure 6A**; **Table 2**). This identifies diabetes, particularly type 2 diabetes, as a very strong risk factor for PMO. Although patients may have normal or even high bone mineral density (BMD), they often have poor bone quality (low bone turnover, abnormal collagen cross-linking, increased microdamage accumulation), leading to increased bone fragility. Hyperglycemia, accumulation of advanced glycation end products (AGEs), and diabetic complications (e.g., nephropathy, vision decline) are factors that may collectively underlie the observed elevated fracture risk.

**Figure 6:**
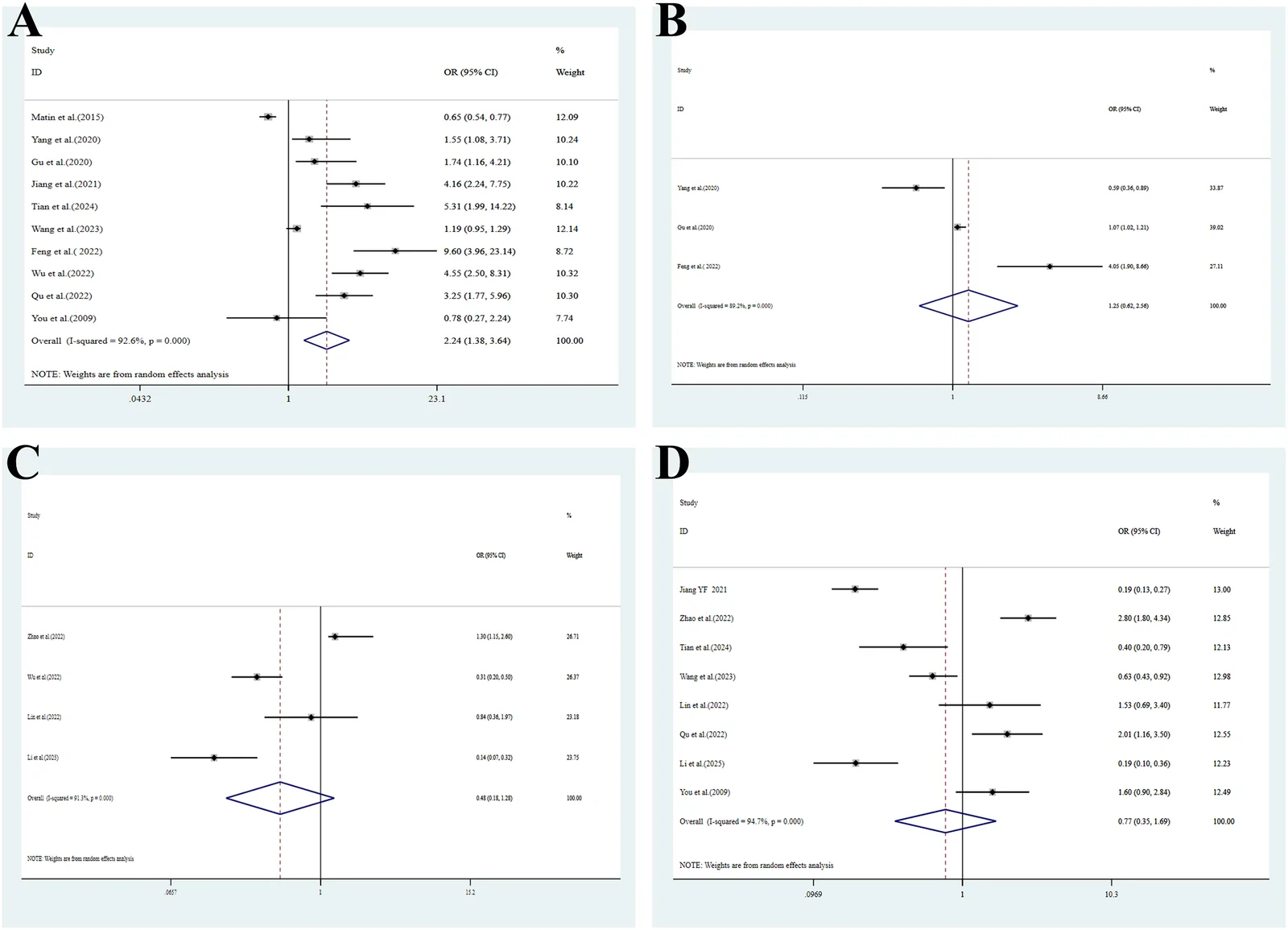
Forest plots of meta-analysis for metabolic, pharmacological, and nutritional factors associated with osteoporosis in postmenopausal women. **(A)** Diabetes mellitus: The overall pooled OR from random-effects meta-analysis was 2.24 (95% CI: 1.38–3.64, p = 0.001), indicating a significant association between diabetes and increased odds of osteoporosis. Heterogeneity was high (I²= 92.6%, p< 0.001). **(B)** Hypertension: The pooled OR was 1.25 (95% CI: 0.62–2.56, p = 0.532), indicating no statistically significant association between hypertension and osteoporosis in the overall analysis. Heterogeneity was very high (I²= 89.2%, p< 0.001). **(C)** Estrogen replacement therapy: The overall pooled OR was 0.45 (95% CI: 0.23–0.90, p = 0.024), suggesting a significant protective effect of estrogen replacement therapy against osteoporosis. Heterogeneity was moderate (I²= 90.4%, p< 0.001). **(D)** Calcium supplementation: The pooled OR was 0.77 (95% CI: 0.35–1.69, p = 0.508), indicating no statistically significant association in the overall analysis between calcium supplementation and osteoporosis risk. Heterogeneity was substantial (I²= 94.7%, p< 0.001).

For diabetes mellitus, substantial heterogeneity was observed (I² > 92.6%, P < 0.001). The binary “yes/no” outcome lacked stratification regarding diabetes type (e.g., type 1 vs. type 2), disease duration, or glycemic control, which limited our ability to explore clinical subgroups; therefore, we performed subgroup analysis stratified by geographic region. This approach partially reduced the variability (I² decreased to 58.3% in East Asia; **Supplementary Figure S1F**), suggesting that regional differences in diabetes diagnostic criteria and screening practices likely contributed to, but did not fully account for, the observed heterogeneity.

We then assessed potential small-study effects. The funnel plot appeared largely symmetric (**Supplementary Figure S2I**), arguing against substantial publication bias. This visual impression was supported by a non-significant Begg’s test (p = 0.655), whereas the Egger’s test was significant (p = 0.021; **Supplementary Table S3**). This dissociation may reflect methodological diversity in defining and reporting diabetes status rather than selective publication.

Finally, leave-one-out sensitivity analysis suggested the robustness of the pooled estimate (**Supplementary Figure S3I**). Therefore, despite persistent heterogeneity likely rooted in the structural limitations of binary diabetes reporting and population-specific diagnostic thresholds, the integrated analysis provides a stable estimate based on currently available evidence.

### 3.16 Hypertension

3 studies (57, 59, 68) investigated the impact of hypertension. High heterogeneity was present (I² = 89.2%, P < 0.001), leading to a random-effects model. The pooled OR was 1.25 (95% CI: 0.62–2.56, P = 0.532) (**Figure 6B**; **Table 2**). The association is not statistically significant. Hypertension and osteoporosis may share some common pathophysiological pathways, such as activation of the renin-angiotensin system and a chronic inflammatory state. However, this analysis could not confirm a significant independent effect after adjusting for other factors, suggesting more studies are needed to clarify their relationship. Nevertheless, given that this conclusion is derived from only three included studies, it should be interpreted with caution when extrapolating to clinical practice, as the limited evidence base precludes definitive clinical recommendations regarding hypertension as a risk factor for PMO.

For hypertension, substantial heterogeneity was also observed (I² = 89.2%, P < 0.001). The binary “yes/no” outcome lacked stratification regarding hypertension stage, duration of disease, or antihypertensive medication use, which limited our ability to explore clinical subgroups. Notably, all included studies examining this factor were derived exclusively from Chinese cohorts, precluding subgroup analysis by geographic region.

Assessment of small-study effects demonstrated a largely symmetric funnel plot (**Supplementary Figure S2J**). This visual impression was corroborated by both non-significant Begg’s (p = 0.602) and Egger’s tests (p = 0.875; **Supplementary Table S3**), arguing against substantial publication bias or methodological asymmetry.

Finally, leave-one-out sensitivity analysis suggested the robustness of the pooled estimate (**Supplementary Figure S3J**). Therefore, despite persistent statistical heterogeneity likely rooted in unmeasured clinical variations (e.g., differences in diagnostic criteria or treatment patterns) within this specific population, the integrated analysis provides a stable estimate based on currently available evidence.

### 3.17 Estrogen replacement therapy

5 studies (61, 64, 69, 70, 74) analyzed the impact of estrogen replacement therapy. High heterogeneity was present (I² = 90.4%, P < 0.001), leading to a random-effects model. The pooled OR was 0.45 (95% CI: 0.23–0.90, P = 0.024) (**Figure 6C**; **Table 2**). This is one of the most effective protective measures, reducing risk by approximately 55%. estrogen replacement therapy directly addresses the core pathogenic mechanism of PMO by supplementing exogenous estrogen, effectively inhibiting bone resorption and preserving bone mass. Its use requires careful assessment of benefits (relief of vasomotor symptoms, osteoporosis prevention) versus risks (e.g., venous thromboembolism, stroke, breast cancer) and is suitable for younger women (typically <60 years or within 10 years of menopause) with clear indications.

For estrogen replacement therapy, substantial heterogeneity was also observed (I² = 90.4%, P < 0.001). The binary “yes/no” outcome lacked stratification regarding treatment duration, dosage, formulation, or timing of initiation, which limited our ability to explore clinical subgroups. Notably, all included studies examining this factor were derived exclusively from Chinese cohorts, precluding subgroup analysis by geographic region.

Assessment of small-study effects demonstrated a largely symmetric funnel plot (**Supplementary Figure S2K**). This visual impression was corroborated by both non-significant Begg’s (p = 1) and Egger’s tests (p = 0.961; **Supplementary Table S3**), arguing against substantial publication bias or methodological asymmetry.

Finally, leave-one-out sensitivity analysis suggested the robustness of the pooled estimate (**Supplementary Figure S3K**). Therefore, despite persistent statistical heterogeneity likely rooted in unmeasured variations in hormone therapy regimens and menopausal management practices within this specific population, the integrated analysis provides a stable estimate based on currently available evidence.

### 3.18 Calcium supplementation

8 studies (61, 64, 65, 67, 70, 71, 74, 75) evaluated the impact of calcium supplementation. High heterogeneity was present (I² = 94.7%, P < 0.001), leading to a random-effects model. The pooled OR was 0.77 (95% CI: 0.35–1.69, P = 0.508) (**Figure 6D**; **Table 2**). No significant protective effect was shown. This may be because the benefit of calcium supplementation is not apparent in populations with adequate intake, or its effect depends on sufficient vitamin D levels for absorption. Furthermore, differences in supplementation dose, duration, and baseline calcium intake across studies may contribute to the heterogeneous results. Calcium and vitamin D supplementation should be considered basic supportive therapy, not a substitute for anti-resorptive or bone-forming medications.

For calcium supplementation, substantial heterogeneity was also observed (I² = 94.7%, P < 0.001). The binary “yes/no” outcome lacked stratification regarding dosage, duration of supplementation, formulation (e.g., carbonate vs. citrate), or adherence levels, which limited our ability to explore clinical subgroups. Notably, all included studies examining this factor were derived exclusively from Chinese cohorts, precluding subgroup analysis by geographic region.

Assessment of small-study effects demonstrated a largely symmetric funnel plot (**Supplementary Figure S2L**). This visual impression was corroborated by both non-significant Begg’s (p = 1) and Egger’s tests (p = 0.577; **Supplementary Table S3**), arguing against substantial publication bias or methodological asymmetry.

Finally, leave-one-out sensitivity analysis suggested the robustness of the pooled estimate (**Supplementary Figure S3L**). Therefore, despite persistent statistical heterogeneity likely rooted in unmeasured variations in supplementation regimens and dietary calcium intake within this specific population, the integrated analysis provides a stable estimate based on currently available evidence.

In summary, this meta-analysis identified a series of factors significantly associated with PMO. Based on their effect sizes (OR) and statistical significance, they can be categorized as follows:

Extremely high-risk factors (OR > 3.0): With ≥2 births (OR = 3.07).

High-risk factors (2.0 ≤ OR < 3.0): Postmenopausal duration >10 years (OR = 2.81), Diabetes mellitus (OR = 2.24).

Moderate-risk factors (1.5 ≤ OR < 2.0): History of previous fracture (OR = 1.86), Lack of exercise (OR = 1.66), Smoking (OR = 1.55).

Low-risk factors (1.0 < OR ≤ 1.5): Age (OR = 1.16), Postmenopausal duration (OR = 1.23), Number of births (OR = 1.32).

Protective factors (OR < 1.0): Higher BMI (OR = 0.80), Older age at menopause (OR = 0.92), Estrogen replacement therapy (OR = 0.45).

Non-significant factors: Family history of osteoporosis, Regular exercise, Hypertension, Calcium supplementation.

These findings provide important evidence-based support for the clinical identification of high-risk postmenopausal women for osteoporosis and the development of targeted prevention and intervention strategies (**Table 3**).

**Table 3:** Summary of significant risk factors for postmenopausal osteoporosis.

Risk factor	OR	95% CI	P-value	Risk level
With ≥2 births	3.07	(2.10–4.50)	<0.001	Extremely High
Postmenopausal duration >10 years	2.81	(2.08–3.78)	<0.001	High
Diabetes mellitus	2.24	(1.38–3.64)	0.001	High
History of previous fracture	1.86	(1.22–2.85)	0.004	Moderate
Lack of exercise	1.66	(1.07–2.56)	0.023	Moderate
Smoking	1.55	(1.24–1.93)	<0.001	Moderate
Number of births	1.32	(1.03–1.69)	0.026	Low
Postmenopausal duration	1.23	(1.13–1.33)	<0.001	Low
Age (per 1-year increase)	1.16	(1.11–1.22)	<0.001	Low
Age at menopause (older)	0.92	(0.89–0.96)	<0.001	Protective
BMI (higher)	0.80	(0.75–0.87)	<0.001	Protective
Estrogen replacement therapy	0.45	(0.23–0.90)	0.024	Protective

Only factors reaching statistical significance (P<0.05) are listed. OR>1 indicates increased risk; OR<1 indicates a protective effect. Non-significant factors (family history of osteoporosis, regular exercise, hypertension, calcium supplementation) are not included.

## 4 Discussion

### 4.1 Principal findings and context

This meta-analysis synthesizes adjusted estimates from 37 observational studies involving 32,366 women to delineate a hierarchy of independent risk factors for PMO (80). We quantified a pooled prevalence of 33.87%, which aligns with mid-range global estimates (20%–40%) and reflects variations in genetics, diet, and BMD screening access across regions (81–83). This high prevalence underscores the epidemic burden of PMO, which drives substantial personal morbidity and socioeconomic costs through fragility fractures (84–87). Our findings provide a robust, multivariate-adjusted framework for clinical risk stratification, moving beyond crude associations to inform targeted prevention strategies (**Table 3**).

### 4.2 Interpretation of key risk factors

#### 4.2.1 Non-modifiable determinants: age, estrogen deficiency, and reproductive history

Consistent with established pathophysiology, advancing age and prolonged estrogen deficiency (postmenopausal duration) were fundamental drivers of PMO risk, with a marked acceleration of risk beyond 10 years postmenopause (27, 88, 89). Reproductive history also emerged as a critical domain. While parity showed a modest linear association, having ≥2 births conferred a three-fold increased risk (OR = 3.07). This likely reflects cumulative maternal skeletal calcium mobilization during pregnancy and lactation, compounded by insufficient inter-pregnancy nutritional recovery (90, 91).

#### 4.2.2 Modifiable lifestyle factors

Lifestyle factors yielded clear, actionable insights. Smoking and lack of exercise were confirmed as significant, modifiable risk factors (92). While the biological rationale for these associations is well-established in the literature (93–95), the more salient contribution of this meta-analysis lies in explaining the inter-study inconsistencies. For smoking, variations in exposure assessment—such as binary versus quantitative definitions of smoking status (e.g., pack-years, duration)—likely introduced clinical heterogeneity across the four included studies.

For physical inactivity, the non-significant result for “regular exercise” (I²=91.3%) was primarily driven by extreme heterogeneity in how studies operationally defined exercise exposure, ranging from structured, quantifiable regimens to binary self-reports without specific frequency, intensity, or duration thresholds. This methodological diversity, rather than a true absence of effect, most plausibly accounts for the divergent findings among included studies. Nevertheless, consensus guidelines strongly advocate for weight-bearing and resistance exercise based on extensive physiological evidence (96, 97).

#### 4.2.3 Anthropometric and clinical predictors

The strong protective association of higher BMI (OR = 0.80) reaffirms the well-recognized “obesity paradox” in bone health (98, 99). However, interpreting this finding requires careful consideration of the substantial heterogeneity observed (I²=85.2%). As detailed in the Results (Section 3.13), geographic subgroup analysis revealed that differences in BMI classification standards and body composition assessment methods across regions—with heterogeneity partially mitigated in West Asian subgroups (I² decreased to 29.9%)—were a major driver of between-study variability. These population-specific differences in measurement approaches, rather than inconsistent true effects, most plausibly explain the divergent estimates. This must be balanced against the increased risk of falls and metabolic complications associated with obesity (100).

Clinically, a history of previous fracture (OR = 1.86) stands out as one of the most powerful predictors, embodying the “fracture cascade” phenomenon. It signifies pre-existing bone fragility and mandates immediate therapeutic intervention to prevent subsequent fractures (101, 102).

#### 4.2.4 Comorbidities — diabetes mellitus

Diabetes mellitus emerged as a high-risk factor (OR = 2.24), independent of BMD. While the biological basis—impaired bone quality and microarchitecture—is increasingly recognized (103, 104), the high heterogeneity (I²=92.6%) across included studies warrants focused interpretation. As reported in the Results (Section 3.15), this variability primarily reflects differences in how studies defined diabetes status (e.g., type 1 vs. type 2, disease duration, glycemic control), with geographic subgroup analysis partially reducing heterogeneity in East Asian cohorts (I² decreased to 58.3%). These methodological differences among studies provide a more plausible explanation for the divergent effect estimates than any true inconsistency in the diabetes-osteoporosis association. Clinically, this underscores the need for BMD-independent assessment tools such as FRAX^®^ with secondary cause adjustment (105, 106).

#### 4.2.5 Pharmacological interventions

Pharmacologically, estrogen replacement therapy demonstrated a strong protective effect (OR = 0.45). However, the high heterogeneity (I²=90.4%) observed across five included studies suggests considerable variability in how this association manifests across populations. As detailed in the Results (Section 3.17), this likely stems from unmeasured variations in hormone therapy regimens (e.g., route of administration, dosage, duration) and menopausal management practices across different cohorts. These clinical and methodological differences among included studies provide a more plausible explanation for the divergent effect estimates than any inconsistency in the underlying biological efficacy of estrogen therapy. This reinforces its role as a highly effective option for osteoporosis prevention in recently menopausal women with significant vasomotor symptoms, following a careful benefit-risk assessment (107, 108). Surprisingly, calcium supplementation alone did not show a significant protective association (OR = 0.77, P = 0.508). This may indicate that its benefit is contingent upon concomitant vitamin D sufficiency for optimal absorption and is most apparent in individuals with baseline deficiency (109, 110). It should be viewed as foundational support rather than monotherapy.

#### 4.2.6 Factors without significant independent association

Notably, family history of osteoporosis did not attain statistical significance as an independent factor. This may reflect inaccuracies in self-reporting, variable definitions, or the polygenic nature of osteoporosis, where the contribution of individual common genetic variants is small and often masked by stronger clinical factors (111, 112). Similarly, hypertension showed no stable association, suggesting any link may be mediated through shared risk factors (e.g., aging, inflammation) rather than a direct causal pathway (113).

### 4.3 Strengths and limitations of the evidence

The principal strength of this study lies in its methodological rigor. By exclusively pooling adjusted estimates from multivariate logistic regression models, we minimized confounding bias and obtained more reliable estimates of independent effects (37). Furthermore, the literature search spanned eight databases—four international (PubMed, Cochrane Library, Embase, Web of Science) and four Chinese regional sources (CNKI, Wanfang, VIP, Sinomed)—and used bilingual search syntax; this design explicitly targeted language bias and allowed inclusion of region-specific PMO epidemiology that would be invisible in an English-only search. The provision of full search strings (**Supplementary Tables S1**, **S2**) makes the process reproducible (114).

Several limitations warrant consideration.

First, the predominance of Chinese cohorts (27/37 studies, 73%) may limit generalizability; while core non-modifiable risk factors align with multinational data (115–117), absolute prevalence and reproductive risk effects may be modulated by ethnicity and diet. Extrapolation to non-East Asian populations should be cautious pending validation (118).

Second, substantial heterogeneity persisted for several factors despite random-effects modeling, likely reflecting clinical or methodological diversity. Notably, differences in osteoporosis diagnostic criteria and BMD assessment methods across studies represent a key source of this variability. While most included studies applied the WHO criterion of T-score ≤ −2.5 at the lumbar spine or hip, some regional studies utilized Z-scores or locally derived normative databases, which may overestimate or underestimate prevalence (119). Furthermore, BMD measurements were obtained at different skeletal sites—including the lumbar spine, femoral neck, total hip, and forearm—each with distinct trabecular bone proportions and susceptibility to age-related artifacts (e.g., aortic calcification affecting lumbar spine T-scores) (120). Variations in dual-energy X-ray absorptiometry (DXA) equipment manufacturers, software versions, and calibration protocols across study sites may have introduced additional measurement error (121). These factors collectively limit the precision of our pooled prevalence estimate and may have contributed to the observed heterogeneity in risk factor associations (122).

Third, residual confounding is possible in observational studies, even with adjustment for multiple covariates, and the cross-sectional or case-control designs of most included studies preclude definitive causal inferences (39).

Furthermore, the lack of standardized definitions for lifestyle-related risk factors across included studies represents a notable limitation. For variables such as lack of exercise, regular exercise, and smoking exposure, operational definitions ranged from explicit, quantifiable criteria to binary self-reports without specific thresholds (123, 124). This definitional variability may have introduced clinical heterogeneity and influenced the pooled estimates, and should be considered when interpreting the results.

Additionally, for some risk factors, statistically significant conclusions were derived from as few as three included studies. While these analyses may still offer exploratory insights into potential associations, the limited number of primary studies reduces statistical power and increases vulnerability to small-study effects. This limitation should be carefully considered, particularly when attempting to translate these specific findings into clinical recommendations without further validation.

Finally, as all included studies were observational in design, the identified associations do not establish causality, and future prospective studies are warranted to confirm these relationships.

### 4.4 Implications for practice and future research

Clinical implications: Our findings provide a robust framework for a stratified, stepwise approach to PMO management in postmenopausal women.

First, risk-stratified screening: Women presenting with high-risk factors (e.g., age > 65 years, postmenopausal duration > 10 years, ≥ 2 births, history of previous fracture, or diabetes mellitus) should be prioritized for immediate DXA screening. For those with moderate-risk factors (e.g., smoking, physical inactivity, or low BMI), a clinical risk assessment using tools like FRAX^®^ (integrating our identified risk factors as secondary causes) should precede DXA, reserving imaging for those exceeding specific threshold probabilities. Notably, a history of previous fracture or diabetes should lower the threshold for initiating specific anti-osteoporotic therapy, even in the context of borderline BMD T-scores, given their strong independent associations with future fracture risk (OR = 1.86 and 2.24, respectively) (17).

Second, tailored pharmacological intervention: The strong protective effect of estrogen replacement therapy (OR = 0.45) reinforces its consideration for younger postmenopausal women (< 60 years or within 10 years of menopause) with significant vasomotor symptoms, provided a careful benefit-risk assessment is conducted. For patients with diabetes or a history of prior fracture, clinicians should prioritize agents with proven efficacy in high-turnover or microarchitecture-compromised bone, such as anabolic agents (e.g., teriparatide) or high-potency antiresorptives (e.g., denosumab), over less potent alternatives (17, 108).

Third, targeted lifestyle modification: Our confirmation of smoking (OR = 1.55) and lack of exercise (OR = 1.66) as significant, modifiable risks provides a clear mandate for behavioral counseling. Clinicians should integrate smoking cessation programs and prescriptions for weight-bearing/resistance exercise into routine care, emphasizing that these interventions are cost-effective strategies to mitigate bone loss and reduce fall risk. (125).

Future research: Priorities include validating these findings in diverse ethnic cohorts, standardizing exposure definitions (e.g., exercise), and integrating these factors into user-friendly clinical risk prediction tools (30). Mechanistic studies on parity- and diabetes-related bone fragility may uncover novel therapeutic targets (126).

## 5 Conclusion

This meta-analysis confirms that PMO is a highly prevalent condition influenced by a constellation of independent factors. It reaffirms the central roles of aging, estrogen deficiency duration, and reproductive history, while providing consistent evidence for the risk associated with smoking, sedentariness, previous fracture, and diabetes, and the protection offered by higher BMI and estrogen replacement therapy. Although high statistical heterogeneity for several factors was addressed using random-effects models, subgroup analyses by geographic region, and leave-one-out sensitivity analyses, these findings—derived predominantly from Chinese cohorts—should be interpreted with caution. The lack of standardized definitions for lifestyle-related risk factors and the persistence of residual clinical heterogeneity may have influenced the pooled estimates. Nevertheless, within these methodological limitations, caution is warranted when extrapolating these findings to clinical practice, particularly for factors supported by a limited number of primary studies. The findings provide a refined, evidence-based framework for identifying high-risk postmenopausal women and enabling more timely, targeted interventions, pending further validation in larger, more diverse cohorts.

## Data Availability

The raw data supporting the conclusions of this article will be made available by the authors, without undue reservation.

## Supplementary material

Supplementary Figure 1 Subgroup analysis of osteoporosis risk factors in postmenopausal women across different geographic regions. Forest plots illustrating the pooled OR with 95% CI for six risk factors: **(A)** Age, **(B)** Years since menopause, **(C)** Regular exercise, **(D)** Body Mass Index (BMI), **(E)** History of previous fracture, and **(F)** Diabetes mellitus. Studies were stratified by geographic region (West Asia, South Asia, East Asia, and Other Regions). The diamond size represents the weight of each subgroup in the random-effects model. The overall pooled OR is represented by the largest diamond at the bottom of each panel. Heterogeneity was assessed using the I-squared statistic. Weights are derived from random effects analysis.

Supplementary Figure 2 Funnel plots for assessing potential publication bias across examined risk factors in the meta-analysis of osteoporosis among postmenopausal women. Panels **(A)** through **(L)** display funnel plots for the following factors, respectively: **(A)** Age, **(B)** Postmenopausal duration, **(C)** Number of births, **(D)** Family history of osteoporosis, **(E)** Regular exercise, **(F)** Lack of exercise, **(G)** BMI, **(H)** History of previous fracture, **(I)** Diabetes mellitus, **(J)** Hypertension, **(K)** Estrogen replacement therapy, **(L)** Calcium supplementation. Each plot positions individual study estimates against their standard error (SE). The dashed lines represent the pseudo 95% confidence limits. The overall symmetrical distribution of data points within the confidence region across all panels suggests a low risk of publication bias. The meta-analytic findings for the examined risk factors are considered robust.

Supplementary Figure 3 Leave-one-out sensitivity analysis for the stability of the pooled effect estimates across examined risk factors of postmenopausal osteoporosis. Panels **(A)** through **(L)** represent sensitivity analyses for the following factors, respectively: **(A)** Age, **(B)** Postmenopausal duration, **(C)** Number of births, **(D)** Family history of osteoporosis, **(E)** Regular exercise, **(F)** Lack of exercise, **(G)** BMI, **(H)** History of previous fracture, **(I)** Diabetes mellitus, **(J)** Hypertension, **(K)** Estrogen replacement therapy, **(L)** Calcium supplementation. Each panel displays the pooled estimate (central vertical line) with its 95% confidence interval from the full meta-analysis, and the recalculated pooled estimates (dots) with confidence intervals (horizontal lines) when each individual study is omitted sequentially. The stability of the point estimates and the consistent overlap of the confidence intervals with the overall estimate across all panels indicate that the meta-analytic results for each factor are not substantially influenced by any single study. The findings are therefore considered robust.
