# Supplementary material for: Prevalence and risk factors of postmenopausal osteoporosis: a systematic review and meta-analysis based on multivariate logistic regression
Source: Front Endocrinol (Lausanne). 2026 Sep 29;17:1950298. doi: 10.3389/fendo.2026.1950298 (PMC13658067; doi:10.3389/fendo.2026.1950298)
Supplement: Supplementary file 1 [file Table1.docx]

**Supplementary material**

**Table S1.** Embase Retrieval strategy.

| No | Query |
| --- | --- |
| #1 | ('postmenopause'/exp OR 'postmenopause') AND [embase]/lim |
| #2 | ('post menopause'/exp OR 'post menopause' OR 'postmenopausal female'/exp OR 'postmenopausal female' OR 'postmenopausal period'/exp OR 'postmenopausal period' OR 'postmenopausal women'/exp OR 'postmenopausal women' OR 'postmenopause'/exp OR 'postmenopause') AND [embase]/lim |
| #3 | ('postmenopausal women'/exp OR 'postmenopausal women' OR (postmenopausal AND ('women'/exp OR 'women' OR 'women'/exp OR women))) AND [embase]/lim |
| #4 | #1 OR #2 OR #3 |
| #5 | ('postmenopause osteoporosis'/exp OR 'postmenopause osteoporosis') AND [embase]/lim |
| #6 | ('osteoporosis, postmenopausal'/exp OR 'osteoporosis, postmenopausal' OR 'post-menopausal bone loss'/exp OR 'post-menopausal bone loss' OR 'post-menopausal osteoporosis'/exp OR 'post-menopausal osteoporosis' OR 'post-menopause bone loss'/exp OR 'post-menopause bone loss' OR 'post-menopause osteoporosis'/exp OR 'post-menopause osteoporosis' OR 'postmenopausal bone loss'/exp OR 'postmenopausal bone loss' OR 'postmenopausal osteoporosis'/exp OR 'postmenopausal osteoporosis' OR 'postmenopause bone loss'/exp OR 'postmenopause bone loss' OR 'type 1 osteoporosis'/exp OR 'type 1 osteoporosis' OR 'type i osteoporosis'/exp OR 'type i osteoporosis' OR 'postmenopause osteoporosis'/exp OR 'postmenopause osteoporosis') AND [embase]/lim |
| #7 | #5 OR #6 |
| #8 | ('risk factor'/exp OR 'risk factor') AND [embase]/lim |
| #9 | ('relative risk'/exp OR 'relative risk' OR 'risk factors'/exp OR 'risk factors' OR 'risk factor'/exp OR 'risk factor') AND [embase]/lim |
| #10 | #8 OR #9 |
| #11 | ('cohort analysis'/exp OR 'cohort analysis') AND [embase]/lim |
| #12 | ('analysis, cohort'/exp OR 'analysis, cohort' OR 'cohort fertility'/exp OR 'cohort fertility' OR 'cohort life cycle'/exp OR 'cohort life cycle' OR 'cohort studies'/exp OR 'cohort studies' OR 'cohort study'/exp OR 'cohort study' OR 'fertility, cohort'/exp OR 'fertility, cohort' OR 'cohort analysis'/exp OR 'cohort analysis') AND [embase]/lim |
| #13 | #11 OR #12 |
| #14 | ('case control study'/exp OR 'case control study') AND [embase]/lim |
| #15 | ('case-control studies'/exp OR 'case-control studies' OR 'case-control study'/exp OR 'case-control study' OR 'control study, case'/exp OR 'control study, case' OR 'matched case control'/exp OR 'matched case control' OR 'matched case control studies'/exp OR 'matched case control studies' OR 'matched case control study'/exp OR 'matched case control study' OR 'case control study'/exp OR 'case control study') AND [embase]/lim |
| #16 | #14 OR #15 |
| #17 | ('cross-sectional study'/exp OR 'cross-sectional study') AND [embase]/lim |
| #18 | ('cross-sectional design'/exp OR 'cross-sectional design' OR 'cross-sectional research'/exp OR 'cross-sectional research' OR 'cross-sectional studies'/exp OR 'cross-sectional studies' OR 'cross-sectional study'/exp OR 'cross-sectional study') AND [embase]/lim |
| #19 | #17 OR #18 |
| #20 | #13 OR #16 OR #19 |
| #21 | #4 AND #7 AND #10 AND #20 |
