# Supplementary material for: Prevalence and risk factors of postmenopausal osteoporosis: a systematic review and meta-analysis based on multivariate logistic regression
Source: Front Endocrinol (Lausanne). 2026 Sep 29;17:1950298. doi: 10.3389/fendo.2026.1950298 (PMC13658067; doi:10.3389/fendo.2026.1950298)
Supplement: Supplementary file 2 [file Table2.docx]

**Supplementary material**

**Table S2.** Pubmed Retrieval strategy.

| No | Query |
| --- | --- |
| #1 | ((((((Postmenopausal women) OR (postmenopause)) OR (post menopause)) OR (postmenopausal female)) OR (postmenopausal period)) OR (postmenopausal women)) OR (postmenopause) |
| #2 | "Osteoporosis, Postmenopausal"[Mesh] |
| #3 | #2 OR #3 |
| #4 | "Risk Factors"[Mesh] |
| #5 | (((((((((((((((((Factor, Risk) OR (Risk Factor)) OR (Population at Risk)) OR (Populations at Risk)) OR (Risk Scores)) OR (Risk Score)) OR (Score, Risk)) OR (Risk Factor Scores)) OR (Risk Factor Score)) OR (Score, Risk Factor)) OR (Health Correlates)) OR (Correlates, Health)) OR (Social Risk Factors)) OR (Factor, Social Risk)) OR (Factors, Social Risk)) OR (Risk Factor, Social)) OR (Risk Factors, Social)) OR (Social Risk Factor) |
| #6 | (((((((((((((((((Factor, Risk) OR (Risk Factor)) OR (Population at Risk)) OR (Populations at Risk)) OR (Risk Scores)) OR (Risk Score)) OR (Score, Risk)) OR (Risk Factor Scores)) OR (Risk Factor Score)) OR (Score, Risk Factor)) OR (Health Correlates)) OR (Correlates, Health)) OR (Social Risk Factors)) OR (Factor, Social Risk)) OR (Factors, Social Risk)) OR (Risk Factor, Social)) OR (Risk Factors, Social)) OR (Social Risk Factor) |
| #7 | #5 OR #6 |
| #8 | #1 AND #4 AND #7 |
| #9 | "Cohort Studies"[Mesh] |
| #10 | ((((((((((((((((((((((((((((((((Cohort Study) OR (Studies, Cohort)) OR (Study, Cohort)) OR (Studies, Concurrent)) OR (Concurrent Study)) OR (Study, Concurrent)) OR (Concurrent Studies)) OR (Closed Cohort Studies)) OR (Cohort Studies, Closed)) OR (Closed Cohort Study)) OR (Cohort Study, Closed)) OR (Study, Closed Cohort)) OR (Studies, Closed Cohort)) OR (Historical Cohort Studies)) OR (Cohort Studies, Historical)) OR (Cohort Study, Historical)) OR (Historical Cohort Study)) OR (Study, Historical Cohort)) OR (Studies, Historical Cohort)) OR (Incidence Studies)) OR (Incidence Study)) OR (Studies, Incidence)) OR (Study, Incidence)) OR (Analysis, Cohort)) OR (Analyses, Cohort)) OR (Cohort Analyses)) OR (Cohort Analysis)) OR (Birth Cohort Studies)) OR (Birth Cohort Study)) OR (Cohort Studies, Birth)) OR (Cohort Study, Birth)) OR (Studies, Birth Cohort)) OR (Study, Birth Cohort) |
| #11 | #9 OR #10 |
| #12 | "Case-Control Studies"[Mesh] |
| #13 | ((((((((((((((((((((((((((((((((((((((((Case-Control Study) OR (Studies, Case-Control)) OR (Study, Case-Control)) OR (Case-Comparison Studies)) OR (Case Comparison Studies)) OR (Case-Comparison Study)) OR (Studies, Case-Comparison)) OR (Study, Case-Comparison)) OR (Case-Compeer Studies)) OR (Studies, Case-Compeer)) OR (Case-Base Studies)) OR (Case Base Studies)) OR (Studies, Case-Base)) OR (Case-Referent Studies)) OR (Case Referent Studies)) OR (Case-Referent Study)) OR (Studies, Case-Referent)) OR (Study, Case-Referent)) OR (Case-Referrent Studies)) OR (Case Referrent Studies)) OR (Case-Referrent Study)) OR (Studies, Case-Referrent)) OR (Study, Case-Referrent)) OR (Case Control Studies)) OR (Case Control Study)) OR (Studies, Case Control)) OR (Study, Case Control)) OR (Matched Case-Control Studies)) OR (Case-Control Studies, Matched)) OR (Case-Control Study, Matched)) OR (Matched Case Control Studies)) OR (Matched Case-Control Study)) OR (Studies, Matched Case-Control)) OR (Study, Matched Case-Control)) OR (Nested Case-Control Studies)) OR (Case-Control Studies, Nested)) OR (Case-Control Study, Nested)) OR (Nested Case Control Studies)) OR (Nested Case-Control Study)) OR (Studies, Nested Case-Control)) OR (Study, Nested Case-Control) |
| #14 | #12 OR #13 |
| #15 | "Cross-Sectional Studies"[Mesh] |
| #16 | ((((((((((((((((((((((((Cross Sectional Studies) OR (Cross-Sectional Study)) OR (Studies, Cross-Sectional)) OR (Study, Cross-Sectional)) OR (Cross-Sectional Survey)) OR (Cross Sectional Survey)) OR (Cross-Sectional Surveys)) OR (Survey, Cross-Sectional)) OR (Surveys, Cross-Sectional)) OR (Surveys, Disease Frequency)) OR (Disease Frequency Survey)) OR (Survey, Disease Frequency)) OR (Disease Frequency Surveys)) OR (Analysis, Cross-Sectional)) OR (Analyses, Cross-Sectional)) OR (Analysis, Cross Sectional)) OR (Cross-Sectional Analyses)) OR (Cross-Sectional Analysis)) OR (Cross Sectional Analysis)) OR (Analyses, Cross Sectional)) OR (Cross Sectional Analyses)) OR (Prevalence Studies)) OR (Prevalence Study)) OR (Studies, Prevalence)) OR (Study, Prevalence) |
| #17 | #15 OR #16 |
| #18 | #11 OR #14 OR #17 |
| #19 | #8 AND #18 |
