# Supplementary material for: Prevalence and risk factors of postmenopausal osteoporosis: a systematic review and meta-analysis based on multivariate logistic regression
Source: Front Endocrinol (Lausanne). 2026 Sep 29;17:1950298. doi: 10.3389/fendo.2026.1950298 (PMC13658067; doi:10.3389/fendo.2026.1950298)
Supplement: Supplementary file 3 [file Table3.docx]

**Table S3.** Publish biased assessments.

| **Outcome** | **P value for Begg test** | **P value for Egger test** |
| --- | --- | --- |
| Age | 0.714 | 0.003 |
| Postmenopausal duration | 0.211 | 0.01 |
| Number of births | 0.322 | 0.611 |
| Family history of osteoporosis | 0.624 | 0.384 |
| Regular exercise | 0.216 | 0.27 |
| Lack of exercise | 0.573 | 0.401 |
| BMI | 0.024 | 0.011 |
| History of previous fracture | 0.404 | 0.065 |
| Diabetes mellitus | 0.655 | 0.021 |
| Hypertension | 0.602 | 0.875 |
| Estrogen Replacement Therapy | 1 | 0.961 |
| Calcium supplementation | 1 | 0.577 |
